# Editorial: Inflammation in the elderly: therapeutic drugs and strategies

**DOI:** 10.3389/fmed.2025.1767912

**Published:** 2026-01-12

**Authors:** Mingbao Lin

**Affiliations:** Institute of Materia Medica, Chinese Academy of Medical Sciences & Peking Union Medical College, Beijing, China

**Keywords:** cellular senescence, gut microbiota, immunosenescence, inflammation clocks, metaflammation, neuroinflammation

The understanding of inflammation in older people has evolved dramatically, moving from a vague concept of “inflammation-aging” to a detailed, mechanistic field with significant clinical implications. Research is now focused on its sources, mechanisms, and consequences, rather than just describing it.

Firstly, cellular senescence and the senescence-associated secretory phenotype (SASP) are the most active area of research. Drugs that clear senescent cells (senolytics like dasatinib, quercetin, fisetin) or suppress SASP (senomorphics) have shown promise in animal models to reduce inflammation and improve healthspan. Early human trials (e.g., for idiopathic pulmonary fibrosis, diabetic kidney disease) are ongoing.

Secondly, immunosenescence is not just a decline, but a dysregulation. The innate immune system shifts from protecting against threat to a hyper-responsive, pro-inflammatory state. The adaptive immune system also shifts toward a pro-inflammatory profile. For example, Th17 cells defend against extracellular bacteria and fungi but can drive autoimmunity and chronic inflammation by producing IL-17, IL-21, IL-22, and TNF-α. And regulatory T-cells (Tregs) maintain immune tolerance, prevent autoimmunity, and resolve inflammation, primarily via producing IL-10, TGF-β, and through cell-contact inhibition. A sustained Th17/Treg imbalance is a hallmark of many chronic inflammatory and autoimmune conditions. When immunosenescence is pending in older people, an imbalance favoring Th17 over Tregs increases the pro-inflammatory Th17 cells relative to anti-inflammatory Tregs, leading to a pro-inflammatory, tissue-damaging state.

Thirdly, Inflammaging is the core driving force for tissue damage and functional impairment. Gut dysbiosis, inflammaging, and various geriatric diseases such as frailty, sarcopenia, and Alzheimer's disease (AD) are forming a widely studied and interrelated pathogenic network. As people age, the diversity of the gut microbiota decreases, and beneficial bacteria (such as those that produce short-chain fatty acids) decrease while potential harmful bacteria (pro-inflammatory bacteria) increase. This process is influenced by diet (such as a lack of fiber), medications (especially antibiotics), reduced activity, and physiological decline. Age-related changes in gut microbiota (loss of diversity, increase in pro-inflammatory pathobionts) compromise intestinal barrier integrity. Microbial products (e.g., Lipopolysaccharide) enter circulation, triggering systemic inflammation via Toll-like receptors (TLR4). Fecal microbiota transplantation from young to old mice reduces inflammation and improves health, highlighting causal links.

Fourthly, nutrient-sensing pathways (like mTOR, AMPK, sirtuins) and metabolic waste products are integrally linked to inflammation. Mitochondrial Dysfunction: release of mitochondrial DNA (mtDNA) into the cytosol acts as a DAMP (Damage-Associated Molecular Pattern), activating the cGAS-STING pathway—a major newly recognized driver of age-related inflammation. Endoplasmic reticulum (ER) stress and unfolded protein response (UPR) can activate inflammatory pathways (e.g., via NF-κB).

In addition, microglial cells (brain's innate immune cells) become primed and dysregulated with age, contributing to neurodegeneration. Peripheral inflammation (e.g., high serum IL-6, TNF-α) is strongly correlated with cognitive decline, depression, and Alzheimer's pathology. The choroid plexus and meningeal lymphatic vessels are now recognized as critical interfaces where peripheral inflammation influences the brain.

Lastly, changes in DNA methylation and histone modification patterns with age can activate inflammatory genes. As one ages, the overall methylation level of the genome decreases, especially in repetitive sequences and intergenic regions. This leads to an increase in genomic instability and may release the inhibition on certain pro-inflammatory genes or transposons, triggering a constitutive immune response similar to viral infection. In the promoter regions of specific genes, especially those related to cell cycle regulation, DNA repair, and tumor suppression, there is an abnormally high methylation and resulting silencing of these genes. This weakens the cell's stress resistance and repair capabilities. In the promoter regions of key pro-inflammatory cytokine genes such as interleukin-1β, interleukin-6, and tumor necrosis factor-α, age-related methylation loss is frequently observed. This makes the chromatin structure of these genes more “open,” making them more accessible for transcription factor binding and activation, thus entering a pro-inflammatory state. In the meantime, the expression of histones in senescent cells decreases, which may affect the structure of nucleosomes and the global gene expression. In summary, age-related epigenetic changes are the “programming code” and “molecular switch” of inflammatory aging. They reshape the way the genome responds to stimuli, locking aging cells and tissues into a chronic, low-level, and self-amplifying inflammatory state, providing the environment for the emergence of various age-related diseases.

Recent research has transformed inflammation-aging from a descriptive phenomenon to a tractable biological process with identifiable pillars: cellular senescence, immune dysregulation, gut-barrier disruption, and metabolic stress. The focus is now on understanding the crosstalk between these pillars and translating this knowledge into geroscience-guided therapies aimed at extending healthspan by targeting the root causes of age-related chronic inflammation. The goal is not simply to suppress inflammation, but to restore immune homeostasis and resilience.

